# Cesarean Scar Pregnancy Treated by Artery Embolization Combined with Diode Laser: A Novel Approach for a Rare Disease

**DOI:** 10.3390/medicina57050411

**Published:** 2021-04-23

**Authors:** Felice Sorrentino, Vincenzo De Feo, Guglielmo Stabile, Raffaele Tinelli, Maurizio Nicola D’Alterio, Giuseppe Ricci, Stefano Angioni, Luigi Nappi

**Affiliations:** 1 Department of Medical and Surgical Sciences, Institute of Obstetrics and Gynaecology, University of Foggia, 71121 Foggia, Italy; felice.sorrentino.1983@gmail.com (F.S.); vincdefeo@fastwebnet.it (V.D.F.); luigi.nappi@unifg.it (L.N.); 2 Institute for Maternal and Child Health IRCCS “Burlo Garofolo”, 34100 Trieste, Italy; giuseppe.ricci@burlo.trieste.it; 3 Department of Obstetrics and Gynecology, Valle d’Itria Hospital, Martina Franca, 74015 Taranto, Italy; raffaeletinelli@gmail.com; 4 Department of Surgical Sciences, Division of Gynecology and Obstetrics, University of Cagliari, 09042 Cagliari, Italy; mauridalte84@gmail.com (M.N.D.); sangioni@yahoo.it (S.A.); 5 Department of Medicine, Surgery and Health Sciences, University of Trieste, 34100 Trieste, Italy

**Keywords:** scar pregnancy, hysteroscopy, uterine artery embolization (UAE), laser

## Abstract

Cesarean scar pregnancy (CSP) is a rare form of ectopic pregnancy which represents a consequence of a previous cesarean section. It is associated with major maternal morbidity and mortality and has potential implications on future fertility. Because of possible serious complications, CSP should be swiftly diagnosed and treated. There is no management protocol for this rare, life-threatening condition, and each patient should be evaluated individually. Several types of conservative treatment have been used to treat cesarean scar pregnancy: dilation and curettage (D&C), excision of trophoblastic tissues, local or systemic administration of methotrexate, bilateral hypogastric artery ligation, and selective uterine artery embolization with curettage and/or methotrexate administration. In our study we present a cesarean scar pregnancy of a 40-year-old woman who was treated with angiographic uterine artery embolization (UAE) followed by hysteroscopic diode laser resection. Our combined UAE–hysteroscopic laser surgery appears to offer an effective, safe, and minimally invasive surgical treatment.

## 1. Introduction

Cesarean scar pregnancy (CSP) is a rare form of ectopic pregnancy (EP) in which the embryo implants and grows inside the myometrium and the fibrous tissue of the previous cesarean scar. Non-tubal EPs account for less than 10% of all EPs, although their overall incidence has been increasing in recent years [1]. Cesarean scar pregnancies are rare, because they account for <1% of ectopic pregnancies with an incidence of 1:2500 [2].

This type of ectopic pregnancy is extremely hazardous due to the risk of trophoblast penetration through the myometrium up to the uterine vessels. That is why patients have a higher risk of life-threating bleeding, hemoperitoneum, uterine rupture, and shock [3].

The diagnosis includes a combination of clinical symptoms, serology, and ultrasound. The most common symptom of CSP is vaginal bleeding, which is often profuse and painless [4]. The detection of serial hCG levels is commonly used to monitor early pregnancies, but ultrasound findings of the gestational sac (GS) are essential. CSP is identified by trans-vaginal ultrasounds (TVUS) that show: (a) empty uterine cavity and cervical canal, (b) a gestational sac located at the anterior wall of the isthmic portion, separated from the endometrial cavity or the fallopian tube in a previous cesarean scar, (c) a gestational sac embedded within the myometrium and the fibrous tissue of a cesarean section scar at the lower uterine segment with an absence of defect in the myometrium between the bladder and the sac, and (d) a high-velocity low-impedance vascular flow surrounding the gestational sac [5].

Depending on the depth of implantation, we can describe two different types of CSPs: type 1, with preferential development of the pregnancy toward the uterine cavity, and type 2, progressing toward the bladder [6]. Early diagnosis is essential in order to allow for conservative medical and surgical treatments. Due to its relative rarity, an efficient treatment for CSP has not been elucidated yet, and therapy has to be tailored to the patients’ clinical presentation. The desire for future fertility, the size and gestational age of the pregnancy, and hemodynamic stability should be considered when determining a treatment plan. A patient who shows signs of hemorrhage or hemodynamic instability will require surgical intervention. This may include laparoscopy, laparotomy, or hysterectomy. In stable patients, various conservative treatment modalities have been reported, including the local or systemic administration of methotrexate (MTX) [7,8], needle aspiration and local MTX [9], uterine curettage [10], resection of CSP through a transvaginal approach [11], laparoscopic uterine scar resection [12], high-intensity focused ultrasound [13], and angiographic uterine artery embolization (UAE) [8,14,15,16,17,18,19,20]. Among conservative treatments, the laparoscopic excision of a scar pregnancy and isthmocele repair with a barbed suture is the only one that will actually fix the isthmocele and prevent a recurrent ectopic within the cesarean scar [21]. 

A relatively new approach is the use of hysteroscopy to remove cesarean scar pregnancies, which offers numerous advantages, like visually directed treatment, reduced operative bleeding, shorter stay, and better patient compliance [3,22,23,24,25,26].

Herein we present our case of a cesarean scar pregnancy of a 40-year-old woman who was diagnosed early with endovaginal sonography and treated with angiographic uterine artery embolization (UAE) followed by hysteroscopic endocervical resection using diode laser.

## 2. Case Presentation

**Figure 1:**
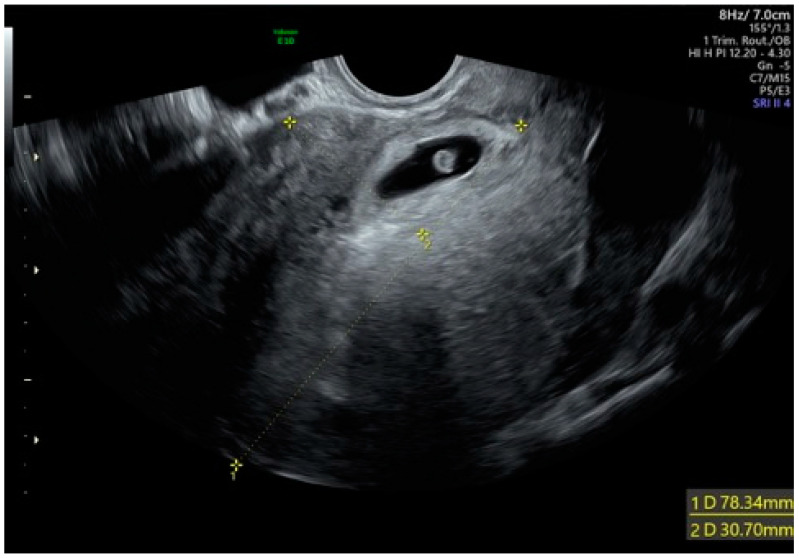
Cesarean scar pregnancy with cardiac activity.

A 40-year-old woman with an amenorrhea of 5.5 weeks was admitted to the Department of Obstetrics and Gynecology of Foggia Hospital with acute onset of poor vaginal bleeding and mild cramping lower abdominal pain. Regarding her obstetric history, she had a normal vaginal delivery and two cesarean sections. The physical examination was remarkable only for the mild lower abdominal tenderness to deep palpation, a small amount of bleeding from the external cervical os, and a mild pain of the uterus. Laboratory data revealed a quantitative serum human chorionic gonadotropin level of 53,539 mIU/mL. A transvaginal ultrasound showed a gestational sac containing a yolk sac and an embryonic pole with cardiac activity (Figure 1), located in the anterior lower uterine wall near a previous cesarean scar. We repeated an ultrasound scan the day after the initial examination and confirmed a persistent gestational sac within the anterior uterine isthmus, close to the anatomic location of the previous cesarean scar (type 1, with preferential development of the pregnancy toward the uterine cavity). There was neither uterine bleeding nor any amount of blood in the abdominal cavity.

The patient was informed about the details of the procedure and signed an informed consent to allow anonymized data collection for research purposes. All procedures performed in the study were in accordance with the ethical standards of the institutional research committee and with the 1964 Helsinki declaration and its later amendments or comparable ethical standards. Approval for the procedure was obtained from the local Ethics and Research Committee. This report follows the Consensus-based Clinical Case Reporting (CARE) guidelines, available through the Enhancing the QUAlity and Transparency Of health Research (EQUATOR) network (https://www.equator-network.org/ accessed on 15 January 2021).

The patient underwent UAE as the first step of treatment. The right femoral artery was cannulated using a flexible angiographic catheter through which the uterine arteries were reached. After selective arteriography, the left uterine artery was totally embolized with a gelatin hemostatic sponge (SPONGOSTAN^TM^). The procedure was repeated on the right uterine artery. The following day we performed a hysteroscopic resection of the ectopic pregnancy. The patient was placed in a dorsal lithotomy position, and a 4-mm continuous-flow office hysteroscope (Bettocchi Office Hysteroscope ‘‘size 4” Karl Storz, Tuttlingen, Germany) with a 2.9-mm rod lens optic was introduced into the cervical canal using the vaginoscopic approach, with no speculum and tenaculum, in an office setting, with no anesthesia or sedation. 

We used a new Dual wavelengths Laser System (Leonardo^®^ Dual 45, Biolitec, Jena, Germany). This highly compact diode laser features the combination of two wavelengths, 980nm and 1470 nm, giving a contemporary absorption in H_2_O and in hemoglobin (Hb) with an excellent ability of hemostasis, cutting, and vaporization, as was previously shown in hysteroscopic and laparoscopic surgery [27,28,29].

**Figure 2:**
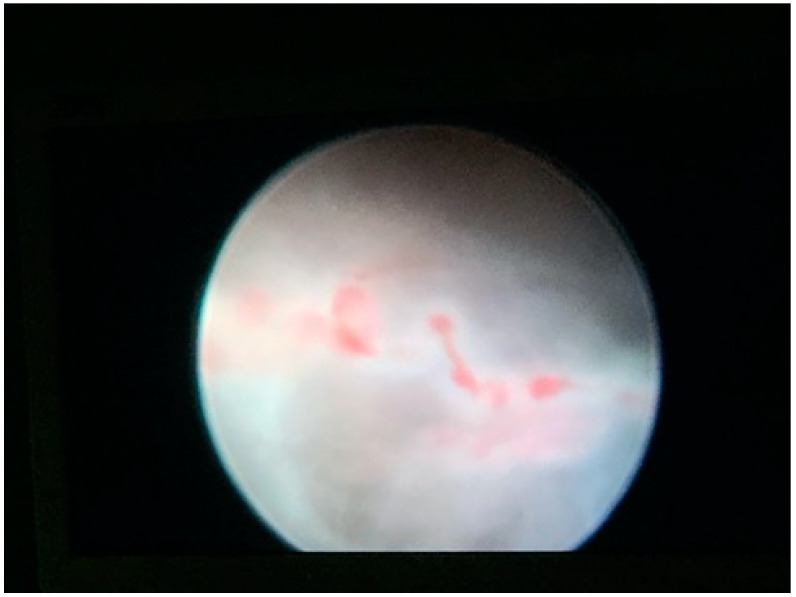
Hysteroscopic view of the umbilical cord and the gestational sac.

**Figure 3:**
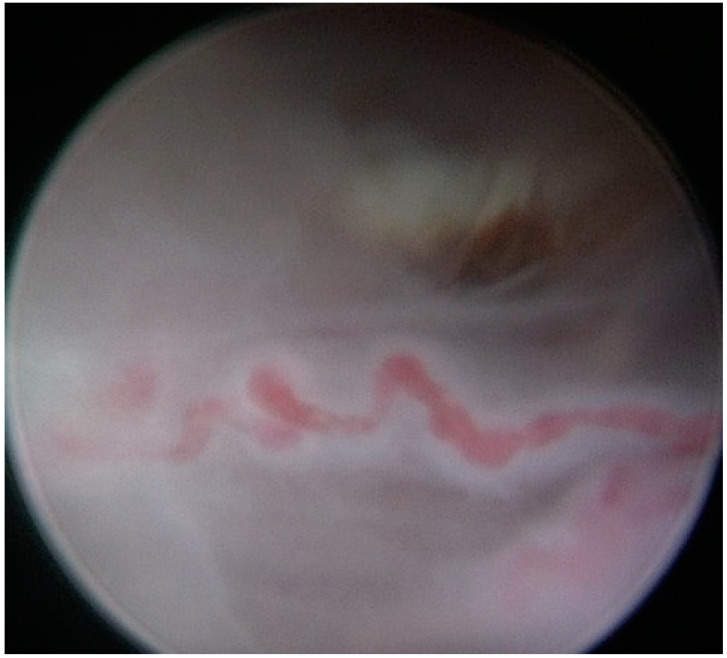
Hysteroscopic view of the umbilical cord.

The gestational sac and the umbilical cord were identified, and the implantation site as the extent of placentation was determined. A conical angled fiber (MyoFiber^®^ CA, IC) with a wider cutting surface was introduced through the operative channel of the hysteroscope, and a laser excision of the ectopic pregnancy near to the implantation site was performed (Figure 2 and Figure 3). The trophoblastic tissue was also removed with the use of the laser and 5 Fr mechanical instruments (crocodile forceps) with excellent control of hemostasis. 

The histological examination confirmed the presence in the endometrium of massive decidual proliferation with necrotic-inflammatory-hemorrhagic phenomena and glandular modifications (Arias Stella phenomenon) and with chorionic villus regression. The patient had an uneventful postoperative recovery and was discharged after three days. Confirmation of the procedure success was obtained from the laboratory data, that showed a negative quantitative serum human chorionic gonadotropin level on the tenth day after the operation. At four weeks follow-up the patient was asymptomatic and under combined oral hormonal contraception.

## 3. Discussion

Cesarean scar pregnancies are rare yet extremely serious conditions, associated with major maternal morbidity and mortality, with potential implications on future fertility. There is no consensus about the best approach to adopt mainly due to a lack of evidence about the best treatment modality after a comparison in large series of clinical cases or randomized studies.

The desired aim of the treatment is to be minimally invasive and to efficiently remove the cesarean scar ectopic pregnancy, assuring minimal morbidity, rapid decline in serum beta hCG up to near normal levels, and a short hospital stay. 

The attitude advocated by many authors is a conservative approach with medical therapy. However, the main disadvantage of medical therapy is the slow resolution of the pregnancy, with the risk of rupture and hemorrhage (hysterectomy may be necessary). For example, a study of 101 individuals with cesarean scar pregnancy treated with an ultrasound-guided methotrexate injection reported a mean time to human chorionic gonadotropin (hCG) normalization of 40 ± 14 days (range: 21–140 days) [30]. UAE has been used to reduce the risk of subsequent hemorrhage in patients who are to undergo conservative surgery. Moreover, UAE plus hysteroscopy demonstrated a success rate of 88% [31], with a very low complication rate [3]. Although there are valid concerns regarding the effects of UAE on women who wish to retain fertility, pregnancy after this procedure is well documented. In general, pregnancy after uterine arterial embolization is possible without significant morbidity or mortality. Furthermore, in the absence of clear data to suggest that UAE has a detrimental effect on reproductive outcomes, the Society of Interventional Radiology (SIR) no longer considers the desire to maintain childbearing potential as a relative contraindication to UAE [32,33]. So, based on these considerations and our experience in office hysteroscopy, we opted for this new approach (UAE plus hysteroscopic endocervical resection using diode laser). Hysteroscopy enables direct visualization and controlled operator movements for evacuation, thus avoiding devastating complications like uterine perforation (the risk of perforation is lower, as no sounding or cervical dilatation is performed) [34].

Moreover it is most of the time a well-tolerated procedure, thus avoiding general anesthesia, and decreasing the costs [35]. Hemostasis can be achieved with electro-coagulation using a wire-loop or roller-ball [36,37]. In our case we used a new device (diode laser) which is a feasible and safe alternative to the scissor, bipolar twizzle, and bipolar or monopolar resectoscope techniques. It demonstrates extreme precision of cutting, controlled power of penetration, a high capacity of hemostasis, the absence of electrical interferences, safety, and a good compliance of patients due to office setting, without the need for cervical dilatation [29]. 

## 4. Conclusions

To the best of our knowledge, there is no case reported about the management of scar pregnancy with our approach. Combined UAE–hysteroscopic laser surgery appears to offer an effective, safe, and minimally invasive surgical treatment to take care of CSP with minimal patient discomfort and optimal recovery time, with low costs in term of operating theatre time, laboratory, and outpatient follow-up. Our technique, although interesting and promising, has to be considered preliminary. One limitation of our procedure could be the risk of recurrent cesarean scar pregnancy, as the isthmocele was not actually excised. Further studies with a larger sample of patients are needed in order to standardize this novel approach.

## Data Availability

The original contributions presented in the study are included in the article, further inquiries can be directed to the corresponding author.
